# Asymptomatic Splenic Infarction Following Total Gastrectomy: A Case Report

**DOI:** 10.70352/scrj.cr.25-0148

**Published:** 2025-08-14

**Authors:** Atsuhito Takagi, Takashi Maeda, Satoshi Kobayashi, Atsushi Sekimura, Takehiro Takagi, Takuya Mishina, Yuya Hibino

**Affiliations:** Department of Surgery, JA Gifu Koseiren Hida Medical Center, Kumiai Kosei Hospital, Takayama, Gifu, Japan

**Keywords:** gastrectomy, extensive splenic infarction, splenic infarction

## Abstract

**INTRODUCTION:**

Splenic infarction is a disease that develops as a result of a thrombotic predisposition. Most areas of malperfusion are usually small and unnoticeable. However, when massive infarction occurs, it is often diagnosed after the onset of clinical symptoms, such as abdominal pain or fever. In contrast, asymptomatic postoperative extensive splenic infarction is occasionally observed. Although conservative management is generally the first-line treatment for splenic infarction, surgical intervention is indicated in cases complicated by splenic abscess or rupture. Some reports suggest that extensive splenic infarction may result in atrophy or complete loss of the spleen over time.

**CASE PRESENTATION:**

A 65-year-old woman presented with chronic epigastric pain and was diagnosed with gastric cancer through upper gastrointestinal endoscopy. The endoscopy revealed an ulcerated mass at the lesser curvature of the middle gastric body, and a biopsy confirmed a moderately differentiated adenocarcinoma. A laparoscopic distal gastrectomy was initially planned. However, the surgical approach was converted to an open total gastrectomy because of the spread of the tumor to the greater curvature of the gastric body and enlargement of the distal lymph nodes of the splenic artery, which were not included in the planned lymphadenectomy. Postoperatively, the patient experienced no abdominal pain or fever. However, on POD 7, blood tests revealed elevated hepatobiliary enzymes, and a contrast-enhanced CT (CECT) scan showed a loss of flow in the splenic artery and vein, leading to a diagnosis of extensive splenic infarction. A follow-up CECT scan 3 months later revealed a notable reduction of the splenic parenchyma over time.

**CONCLUSIONS:**

This is a rare case of asymptomatic, extensive splenic infarction incidentally diagnosed following total gastrectomy and successfully treated with conservative management.

## Abbreviations

CECT
: contrast-enhanced CT
OPSI
: overwhelming post-splenectomy infection

## INTRODUCTION

Splenic infarction is caused by infectious endocarditis, chronic myeloid leukemia, post-traumatic injury, and thrombophilia. Patients with splenic infarction typically present with epigastric pain or fever^1)^; hence, asymptomatic cases are rare. Some reports have classified splenic infarction as partial or extensive based on the extent of the infarcted region. Asymptomatic, extensive splenic infarction following perisplenic surgery is extremely rare. Although conservative management is generally the first-line treatment for common cases, surgical intervention is indicated for cases complicated by splenic abscess or rupture. Additionally, there have been reports of splenic infarction with subsequent atrophy or loss of the spleen over time. Here, we present a rare case of asymptomatic, extensive splenic infarction following gastrectomy, highlighting its clinical course, management, and implications for postoperative care.

## CASE PRESENTATION

**Fig. 1:**
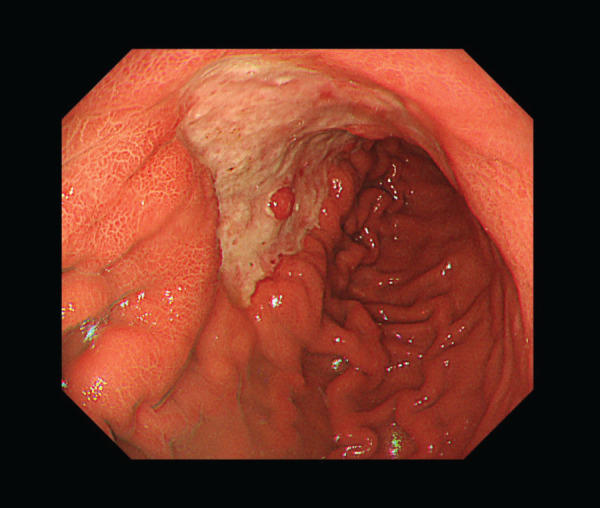
Upper gastrointestinal endoscopy revealed an ulcerated infiltrative lesion centered on the lesser curvature of the gastric angle.

**Fig. 2:**
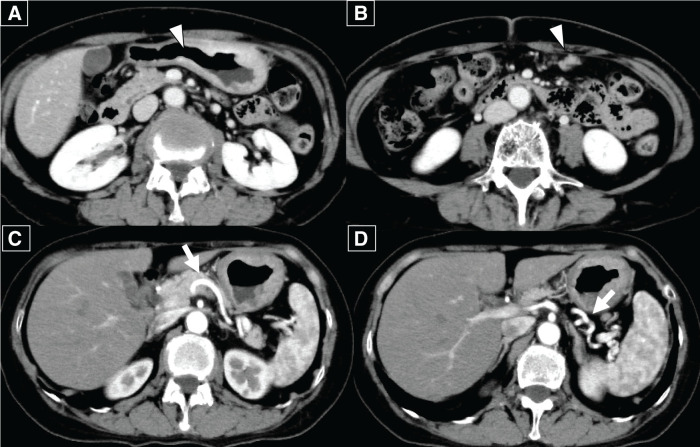
(**A**, **B**) Preoperative contrast-enhanced abdominal CT scan showed wall thickening with enhancement at the gastric angle and an enlarged lymph node along the greater curvature (white arrowhead). (**C**, **D**) A highly tortuous splenic artery was observed (white arrow).

**Fig. 3:**
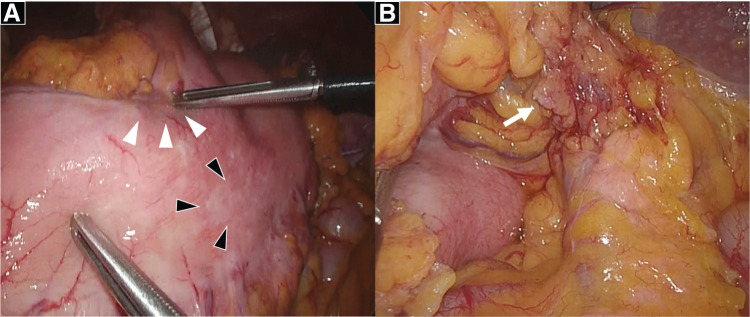
(**A**) The primary lesion was partly exposed on the serosal surface (white arrowheads), with white discoloration suspected to be subserosal tumor invasion (black arrowheads). (**B**) Enlarged distal lymph nodes (#11d) along the splenic artery trunk were observed (white arrow).

A 65-year-old woman presented with chronic epigastric pain and was diagnosed with gastric cancer through upper gastrointestinal endoscopy. Upper gastrointestinal endoscopy revealed an ulcerated mass in the lesser curvature of the middle gastric body (**Fig. 1**). Laboratory tests did not reveal any significant findings. Contrast-enhanced CT (CECT) revealed wall thickening at the lesser curvature and lymph node enlargement at the greater curvature; no distant metastases were detected (**Fig. 2A**–**2D**). Biopsy confirmed a moderately differentiated adenocarcinoma in the tumor specimen, and laparoscopic distal gastrectomy was planned. We initially attempted to perform laparoscopic distal gastrectomy using a 5-port procedure, but we had to convert to open total gastrectomy because of edematous changes observed in the gastric wall extending to the greater curvature (**Fig. 3A** and **3B**). Lymph node dissection was performed in accordance with the Japanese Gastric Cancer Treatment Guidelines,^2)^ implementing spleen-preserving D2 lymphadenectomy for total gastrectomy. Dissection along the splenic artery was conducted from the proximal to distal portion, with complete removal of stations 11p and 11d lymph nodes. The periarterial nerve plexus was preserved throughout the procedure. Methodologically, direct manipulation of the artery was avoided; instead, the surrounding tissues were grasped during dissection. For tissue transection, electrocautery and an ultrasonic coagulating cutting device (Harmonic 1100; Ethicon Endo-Surgery, Cincinnati, OH, USA) were used. The posterior gastric artery, which originated from the splenic artery, was ligated and divided at a location that preserved the integrity of the main arterial trunk. As no significant lymphadenopathy was observed at the splenic hilum, only sampling of station 10 lymph nodes was performed rather than complete dissection. Of particular anatomical note in this case, the patient exhibited pronounced tortuosity of the splenic artery, which formed a distinctive loop-like configuration. During lymphadenectomy along the superior border of the pancreas, the dissection of the splenic vein was limited to partial exposure of its superior aspect. The splenic artery coursed ventral to the splenic vein, forming a loop near the pancreatic body and tail before entering the splenic hilum. In contrast, the splenic vein ascended from the splenic hilum, passed dorsal to the splenic artery, and, unlike the splenic artery, did not form a loop but ran straight to join the superior mesenteric vein. The remaining esophagus and small intestine were reconstructed using Roux-en-Y reconstruction. After surgery, histopathological examination revealed a poorly differentiated adenocarcinoma arising from the middle body of the stomach on the lesser curvature, measuring 130 × 60 mm. There were 31 metastatic nodes, and the tumor was classified as Stage IIIC (T4aN3M0) according to the 8th edition of the tumor-node-metastasis classification. The tumor extensively infiltrated the subserosal layer and spread beyond what was macroscopically visible. Resection margins were negative.

The patient received standard postoperative management. Pain control was achieved using a combination of epidural anesthesia with 0.16% ropivacaine and intravenous (IV) patient-controlled analgesia containing fentanyl (0.5 mg/50 mL), both of which were discontinued on POD 3. Until POD 3, additional IV nonsteroidal anti-inflammatory drugs (NSIDs) were required for pain control; however, no further analgesics were needed thereafter. Subsequently, pain was managed with oral acetaminophen (1200 mg/day) and a diclofenac sodium transdermal patch (150 mg/day).

**Fig. 4:**
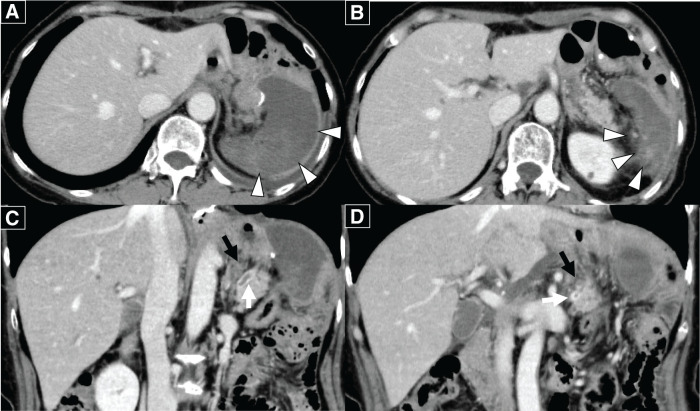
(**A**, **B**) Contrast-enhanced abdominal CT scan showed poor perfusion throughout the splenic parenchyma. Slight contrast enhancement in the splenic parenchyma remains (white arrowheads). (**C**, **D**) A thrombus was noted in the splenic vein (white arrow) and in the tortuous segment of the splenic artery near the pancreatic body (black arrow).

**Fig. 5:**
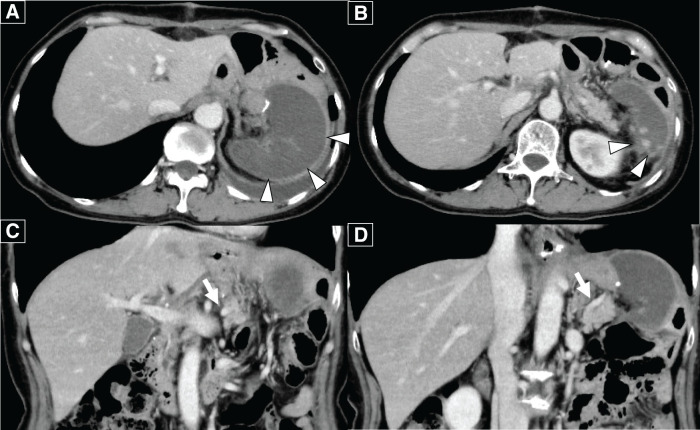
(**A**) As indicated by the white arrowheads, the splenic parenchyma was slightly reduced in size. (**B**) In the splenic hilum (white arrowheads), partial enhancement of the splenic parenchyma was observed. (**C**, **D**) The splenic artery thrombus persisted, but the splenic vein thrombus had resolved, as shown by the white arrow.

**Fig. 6:**
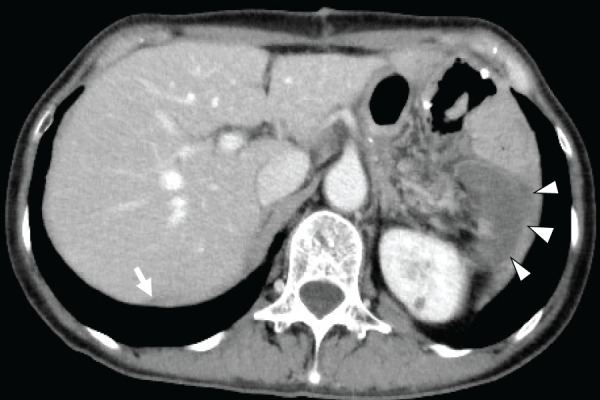
As indicated by the white arrowhead, the splenic parenchyma was notably reduced in size.

On POD 7, scheduled laboratory tests revealed elevated hepatobiliary enzymes, including total bilirubin 1.53 mg/dL, alanine aminotransferase 110 U/L, aspartate aminotransferase 102 U/L, γ-glutamyl transpeptidase 237 U/L, and alkaline phosphatase 148 U/L. Additionally, the D-dimer level was elevated at 9.3 μg/mL. An urgent CECT scan revealed thrombosis in the splenic artery and vein, with interruption of blood flow within the splenic parenchyma (**Fig. 4**). The splenic artery thrombus formed slightly distal to the point where the pancreas transitions from the body to the tail. It was located on the proximal side where the artery loops. Similarly, the venous thrombus originated at the splenic hilum and extended continuously to the vascular loop formed by the splenic artery, observed at the same level as the loop’s origin (**Fig. 4C** and **4D**). The thrombus was confined to the splenic vein. No extension into the superior mesenteric or portal vein was observed. Despite these findings, the patient did not complain of any symptoms, such as abdominal pain or fever. As the splenic parenchyma exhibited extensive ischemia and there was a potential risk of hemorrhage upon recanalization of the splenic artery, and given that the patient remained asymptomatic, neither thrombectomy nor emergent splenectomy was deemed necessary. In light of the serous nature of the intraperitoneal drain output, the elevated D-dimer level indicating a prothrombotic state at the time of splenic infarction, and the potential risk of portal vein thrombosis due to thrombus propagation or extension, the benefits of therapeutic intervention were weighed against the risk of postoperative bleeding. Following this assessment, anticoagulant therapy was initiated. Heparin anticoagulant therapy (15000 U/day) was initiated on the same day. On POD 14, a repeat CECT scan showed a resolution of splenic vein thrombosis, although the thrombus remained in the splenic artery (**Fig. 5**). As the patient’s condition was considered stable, IV heparin was switched to oral edoxaban (30 mg/day) on POD 14, and outpatient follow-up was planned. The patient was discharged on POD 17, and a follow-up CECT scan 3 months later revealed a notable reduction of the splenic parenchyma over time (**Fig. 6**).

Extensive splenic infarction without blood flow to the splenic parenchyma was considered equivalent to splenectomy, resulting in the loss of splenic function. To prevent overwhelming infections after splenectomy, we administered vaccines against pneumococcal and meningococcal diseases. Postoperative adjuvant chemotherapy was initiated after vaccination and remains ongoing. Follow-up CT performed 3 months postoperatively showed no change in the thrombus of the splenic artery, and the D-dimer level had normalized without worsening of the splenic vein thrombus; therefore, oral edoxaban was discontinued.

## DISCUSSION

Several conditions can cause splenic infarction, including infectious endocarditis, chronic myeloid leukemia, post-traumatic injury, and thrombophilia.^1)^ Additionally, some surgical procedures, such as sleeve gastrectomy or pancreatectomy, have been associated with splenic infarction.^3–6)^ The extent of ischemia in splenic infarction varies widely. Blood flow to the spleen is maintained by multiple pathways, and there is a wide range of conditions that can occur, from partial to extensive infarction. In cases like the present one, where contrast-enhanced CT shows loss of enhancement across the entire spleen, we refer to this condition as extensive splenic infarction. The splenic artery arises from the celiac trunk and gives off branches not only to the spleen but also to the stomach and pancreas.^7,8)^ In cases where total gastrectomy has been performed, all gastric branches are typically ligated, eliminating the possibility of gastric-derived collateral circulation. Under such circumstances, if the main trunk of the splenic artery becomes occluded, extensive splenic infarction can be expected due to the lack of an alternative blood supply. While there are multiple reports of splenic infarction after gastrectomy, the majority involves partial infarction of the spleen, and cases of extensive infarction remain relatively rare.^9,10)^ Typical symptoms of splenic infarction are epigastric pain and fever,^1)^ with only a few patients being asymptomatic. Our case was extremely rare because the spleen was extensively ischemic on imaging, while the patient had no symptoms. Regardless of the etiology or extent of infarction, conservative treatment is generally the first-line therapeutic approach for splenic infarction. However, the need for anticoagulant therapy and vaccination remains underexplored. When infectious symptoms such as abdominal pain or fever are present, early surgical intervention is required to manage conditions such as splenic rupture or abscess.

“Postoperative and extensive” splenic infarction is an extremely rare condition, with only 3 similar cases reported in addition to ours. One case was reported in English and listed in PubMed, while the remaining were reported in Japanese and listed in Ichusi, a Japanese article database. These cases, along with the present case, are summarized in **Table 1**.^6,11,12)^ All reported patients were female, with a median age of 61 years (range: 57–69 years old). In other reported cases, left hemicolectomy, distal gastrectomy, and laparoscopic spleen-preserving distal pancreatectomy were performed. Only our patient was asymptomatic, whereas the other patients had abdominal pain as the main complaint when splenic infarction was diagnosed. The median time to detection of splenic infarction was 8 days after surgery (range: 6–15 days), and the median hospital stay was 43 days (range: 17–64 days). All patients were managed conservatively and discharged without any interventional procedures. Additionally, the patients underwent postoperative CT scans, which showed either a reduction in spleen size or extensive resolution of the infarcted spleen.

**Table 1:** Previous case reports of postoperative extensive splenic infarction

No.	Author	Age	Gender	Primary disease	Surgical procedure	Symptom	Onset date	Initial treatment	Length of hospital stay	Splenic atrophy
1	Matsuoka et al.	57	Female	GC	DG	Abdominal pain	POD 6	Conservation	61	Atrophic
2	Sakabe et al.	57	Female	CRC	LHC	Abdominal pain	POD 9	Conservation	64	Disappearance
3	Kimura et al.	69	Female	MCN	LSPDP	Abdominal pain	POD 15	Conservation	25	Atrophic
4	Takagi et al.	65	Female	GC	TG	No symptom	POD 7	Conservation	17	Atrophic

CRC, colorectal cancer; DG, distal gastrectomy; GC, gastric cancer; LHC, left hemicolectomy; LSPDP, laparoscopic spleen-preserving distal pancreatectomy; MCN, mucinous cystic neoplasm; TG, total gastrectomy

We evaluated potential etiologies of splenic infarction following total gastrectomy. The patient had no significant medical history, including hypertension, diabetes mellitus, or dyslipidemia. Additionally, non-contrast CT revealed no evidence of arterial calcification that would suggest underlying atherosclerotic disease. Given that the gastric branches had been surgically divided during the procedure, the infarction likely resulted from occlusion of the main splenic artery trunk. Although meticulous attention was paid to surgical technique, we cannot exclude the possibility of inadvertent direct manipulation of the artery leading to intimal injury. Furthermore, management of arterial branches, such as the posterior gastric artery, may have induced stenosis of the main splenic arterial trunk. A notable anatomical feature in this case was the pronounced tortuosity of the splenic artery. While the periarterial nerve plexus was preserved, the lymphadenectomy procedure inevitably compromised supporting perivascular tissues. Consequently, the tortuous artery may have developed acute angulation and subsequent occlusion without adequate surrounding structural support. Postoperative thrombosis formation remains a possible etiology; however, considering the temporal relationship and radiological findings, mechanical factors related to intraoperative arterial manipulation appear to be the most plausible explanation for the development of splenic infarction in this patient. Additionally, splenic vein thrombosis extended from the splenic hilum to the level corresponding to the origin of the splenic artery loop. Matsumoto et al.^13)^ investigated the risk factors for splenic vein thrombosis following partial splenic embolization and demonstrated that a large infarcted splenic volume may contribute to the development of splenic or portal vein thrombosis. We considered that, in our patient, postoperative inflammatory spread after lymphadenectomy, combined with arterial occlusion caused by tortuous vessels, led to reduced venous outflow from the splenic parenchyma, resulting in venous stasis and subsequent splenic vein thrombosis.

In our case, there was a temporary increase in platelet count, which could have led to thrombophilia. However, antiplatelet therapy was not initiated because the platelet count stabilized before it reached abnormal levels. Anticoagulants were administered to prevent further thrombotic progression. The splenic artery thrombosis showed no extension, the splenic vein thrombosis resolved, and there was no propagation to the superior mesenteric or portal vein. Regarding anticoagulation therapy for splenic vein thrombosis, there are reports of its efficacy, and it was considered an appropriate treatment.^14,15)^

There have been reports of splenic infarction leading to atrophy and eventual loss of the spleen.^11,12)^ However, splenic function following postoperative extensive splenic infarction has not yet been reported. Patients who undergo splenectomy experience a decrease in unswitched memory B cells, resulting in a poor immune response to encapsulated bacteria.^16)^ These patients are at risk of overwhelming post-splenectomy infection (OPSI). Although the incidence rate is low, the mortality is up to 50%.^17)^ Some studies have reported that residual splenic function in cases undergoing splenic artery embolization for traumatic splenic injury can be evaluated by measuring blood cell counts, immunoglobulin levels, T cells, B cells, natural killer cells, and Howell–Jolly bodies.^18)^ Furthermore, Lammers et al. demonstrated, using 99mTc-labeled autologous erythrocyte scintigraphy, that splenic function could be partially preserved in patients after splenectomy because of the development of splenosis.^19)^ Crooker et al. focused on the relationship between remnant spleen volume and splenic function.^20)^ They stated that vaccination for OPSI prevention is necessary in cases with 50% infarction of the splenic parenchyma. Additionally, it has been reported that vaccination against pneumococcal disease is beneficial for patients with solid tumors receiving chemotherapy.^21)^ Based on this, vaccination was recommended for our patient, who was scheduled to receive adjuvant chemotherapy. Vaccination in patients with splenic hypofunction due to extensive splenic infarction is supported by some evidence; however, the therapeutic effect of anticoagulation in improving blood flow is uncertain. Therefore, gaining therapeutic experience in managing patients with extensive splenic infarction is necessary.

## CONCLUSIONS

This report describes a case of asymptomatic extensive splenic infarction following gastrectomy. Extensive splenic infarction is associated with significant atrophy or extensive loss of the spleen, resulting in impaired splenic function. Therefore, vaccination before the initiation of adjuvant chemotherapy may be beneficial following extensive splenic infarction.

## DECLARATIONS

### Funding

None.

### Authors’ contributions

AT, TM, SK, AS, TT, TM, and YH contributed to the diagnosis and treatment of the patient and wrote the first draft of the manuscript.

All authors have read and approved the final manuscript.

### Availability of data and materials

The datasets used in this study are available from the corresponding author upon reasonable request.

### Ethics approval and consent to participate

This case report was approved by the Department of Surgery, JA Gifu Koseiren Hida Medical Center, Kumiai Kosei Hospital (No. K06-08).

### Consent for publication

Written informed consent was obtained from the patient for the publication of this case report and any accompanying images.

### Competing interests

The authors declare that they have no competing interests.
